# Separation and Recovery of Gold(III), Palladium(II) and Platinum(IV) by Solvent Extraction Using a New β-Diketone Derivative from Acidic Solutions

**DOI:** 10.3390/ma14164436

**Published:** 2021-08-08

**Authors:** Elzbieta Radzyminska-Lenarcik, Ilona Pyszka, Artur Kosciuszko

**Affiliations:** 1 Faculty of Chemical Technology and Engineering, UTP University of Science and Technology, Seminaryjna 3, 85-326 Bydgoszcz, Poland; Ilona.Pyszka@utp.edu.pl; 2 Faculty of Mechanical Engineering, UTP University of Science and Technology, Al. Prof. S. Kaliskiego 7, 85-796 Bydgoszcz, Poland; artkos@utp.edu.pl

**Keywords:** solvent extraction, e-waste, precious metal separation, palladium, platinum, gold

## Abstract

This study indicates that a new amine derivative of β-diketone (EDAB-acac) can be successfully used in an acidic medium (HCl) to separate a mixture containing Au(III), Pd(II), and Pt(IV) ions using solvent extraction. The study was conducted in single and ternary model solutions. The impact of acid concentration and the type of solvent (toluene, chloroform, methylene chloride, 2-ethylhexanol) on separation efficiency was discussed. It has been shown that increasing the HCl concentration in the aqueous phase does not favor extraction. In contrast, solvents with high donor numbers (methylene chloride, 2-ethylhexanol) increase both the extraction percentage of Pd and Au as well as the separation coefficients of Pd in relation to Au and Pt. The palladium(II) and gold(III) (which form 4-coordinated planar [MCl_4_]^2−^ complexes) are extracted most efficiently, Pd(II) (87–93%) and Au(III) (56–62%). The stripping of Au(III), Pd(II), and Pt(IV) ions from the EDAB-acac-methylene chloride phase was also investigated using 0.5 M ammonia aq., mineral acid (5 M HCl, 5 M HNO_3_), 0.1 M thiourea in HCl and 0.5 M ammonium thiocyanate. A 3-step stripping process was proposed for the recovery of Pd(II), Au(III), and Pt(IV) from the Pd-Au-Pt mixture in the EDAB-acac-methylene chloride system. In the first stage, the aqueous phase is treated with 5 M HNO_3_ (Pt separation), followed by the application of 0.5 M ammonia (Pd separation) and, finally, 0.1 M thiourea in HCl (Au separation). The solvent extraction with EDAB-acac in acidic medium (HCl) can be used for separation of Pd(II) and Au(III) ions from e-waste leach solutions.

## 1. Introduction

Electrical and electronic equipment has become ubiquitous and the consumption of such goods is increasing every year. The growing demand is accompanied by the increase in the flow of electro-waste (e-waste), which is among the fastest-growing ones in the world [1,2]. According to the latest UN report, in 2019, the world generated about 53.6 million t of electronic waste, with 7.3 kg per capita.

The increasing demand for precious metals is driven by the growing development of advanced technology and electronics. Precious metals are considered strategic metals due to their unique properties and lack of possibility to substitute. Since platinum metals (e.g., Pd, Pt) are among the rarest elements in nature and because of the depleting resources of gold deposits, the process of producing pure precious metals is expensive and technologically difficult. It is, therefore, necessary to recover them both from industrial waste and used products. The high price of platinum ($1203/oz), palladium ($2785/oz) and gold ($175/oz) on the market (BASF Catalysts—Metal Prices) [3] further encourages the search for new ways to recover these metals from industrial waste (e.g., from galvanic and tailings effluents, mining waste heaps, as well as from electronic and electrical waste).

The recycling of electrical and electronic scrap is most justifiable not only for environmental reasons but also for economic reasons due to the possibility of recovering valuable components [4,5].

Precious metals in particular are valuable components. The electronics industry uses about 300 t of gold per year (integrated circuits, contacts), which represents about 9% of the annual production of this metal. With gold contents of 300–350 g Au/t in mobile phones and 200–250 g Au/t in computer components, electronic scrap is a much richer carrier of precious metals compared to primary sources (about 5 g Au/t ore) [6].

In terms of materials, waste electronic equipment is a mixture of various metallic components (including Zn, Al, Ni, Fe, Ga, Se, and In), noble Au, Ag, Pd, Cu, and Pt, hazardous substances (Hg, Be, Pb, As, Cd, and Sb), glass ceramics, and plastics [6,7].

The high content of Al, Fe, Ni, Cu, and Pb, as well as Au, Ag, Pd, and Pt in e-waste makes it a source for metal recovery. According to References [1,8], the metal content of e-waste varies considerably depending on its type (Table 1, Table 2 and Table 3).

The typical metal content in a printed circuit board (PCB) is shown in Table 2 and Table 3 [1].

Although iron is dominant in terms of mass share (Table 1 and Table 2), the value of e-waste is determined by the precious metal content [9]. A seemingly low concentration of these metals in electronic equipment units (less than 0.5%), in terms of global sales, represents a significant share of PGM (precious group metals) production. Referring only to mobile phones and computer equipment, the shares are 4% of world Au production, 10% of Ag, 7% of Pt, and 48% of Pd, respectively.

**Figure 1:**
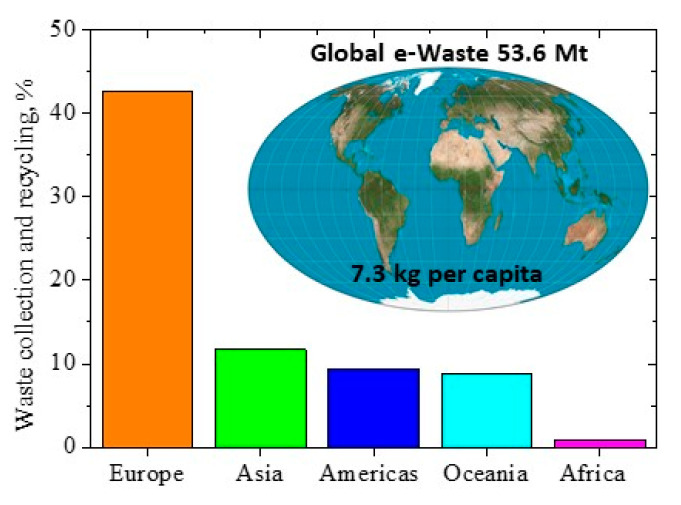
Global e-waste collection and recycling rates. Figure was made by authors.

In 2019, formal documented collection and recycling amounted to 9.3 Mt, or 17.4% of the e-waste generated. This has increased by 1.8 Mt since 2014, an annual increase of almost 0.4 Mt. However, the total amount of e-waste generated increased by 9.2 Mt, with an annual increase of almost 2 Mt. Therefore, recycling efforts have not kept pace with the global increase in e-waste. It is estimated that in 2030 the amount of e-waste generated will exceed 74 Mt [10]. The statistical data on the collection and recycling rate in the world are presented in Figure 1.

Less than 40% of all e-waste in the EU is recycled. Recycling practices vary across the EU. In 2017, Croatia recycled 81% of all electronic and electrical waste, compared to 21% in Malta [8,11].

Pyrometallurgy and hydrometallurgy are two routes that are typically used to recover valuable metals. The traditional methods used to recover metals from electronic waste are pyrometallurgical methods (remelting in furnaces, sintering, melting, high-temperature gas phase reactions) [12,13]. However, in recent decades, the recovery of metals from e-waste via hydrometallurgical methods has been the area of the most intense research [14,15]. These methods are more accurate and do not require complex and expensive equipment [16]. The main steps of hydrometallurgical methods are the rinsing operations of solid electronic waste with acids or lyes [17]. The resulting solutions are then subjected to separation and purification (extraction, adsorption, or ion exchange) [18,19].

Solvent extraction (SX) is one of the hydrometallurgical methods of great importance in the production of metals. The process has been in use for many years but still has great potential for future applications [20]. Many different extractants have been studied for PGM extraction, including quaternary ammonium salts [21,22,23,24,25], tertiary amide extractant [26], piperidine-based extractants [27], pyridine derivatives [28,29], hydroxyoximes [30], trialkylphosphine oxides [31], crown ethers [32], and β-diketone derivatives [33,34,35]. The separation of metal ions via solvent extraction depends mainly on the type of complexing reagent (extractant) and the effective solvent. Therefore, new complexing reagents are being sought to achieve the efficient separation of these ions from aqueous solutions.

The authors of this study decided to present the results of research on the usefulness of an amine derivative of β-diketone (ethylenodiamino-bis-acetylacetone (EDAB-acac)) in the separation of gold(III), platinum(IV), and palladium(II) ions from model solutions by SX in acidic medium.

Previously, EDAB-acac was used for Zn separation from a ternary Zn-Cr-Ni mixture [36] and a five-component Zn-Co-Ni-Cu-Cd mixture [37]. The zinc separation processes from these mixtures were carried out in a slightly alkaline (ammonia) environment with the use of polymer inclusion membranes.

When projecting the research described in this paper, the authors assumed that the use of differences in the complexing properties of Pd(II), Pt(IV), and Au(III) ions (i.e., differences in the formation of stable chelates with EDAB-acac in the HCl medium) will enable the separation of at least one or two examined metals.

## 2. Materials and Methods

### 2.1. Reagents

Commercial AuCl_3_ (Sigma-Aldrich, Poznan, Poland), palladium chloride PdCl_2_ (99%, Pol-Aura, Zabrze, Poland), PtCl_4_ (Pol-Aura, Zabrze, Poland), HNO_3_ (65%, Avantor Performance Materials Poland S.A., Gliwice, Poland), HCl (35%, Chempur, Piekary Slaskie, Poland), NH_3aq_ (25%, Chempur, Piekary Slaskie, Poland), NH_4_SCN, and thiourea (both from Chempur, Piekary Slaskie, Poland) were used to prepare the initial solutions. Methylene chloride (Fluka, Busch, Switzerland), 2-ethylhexanol (99.6%, Sigma-Aldrich, Poznan, Poland), chloroform, and toluene (both from Chempur, Piekary Slaskie, Poland) were used as extractant’s diluents.

**Figure 2:**
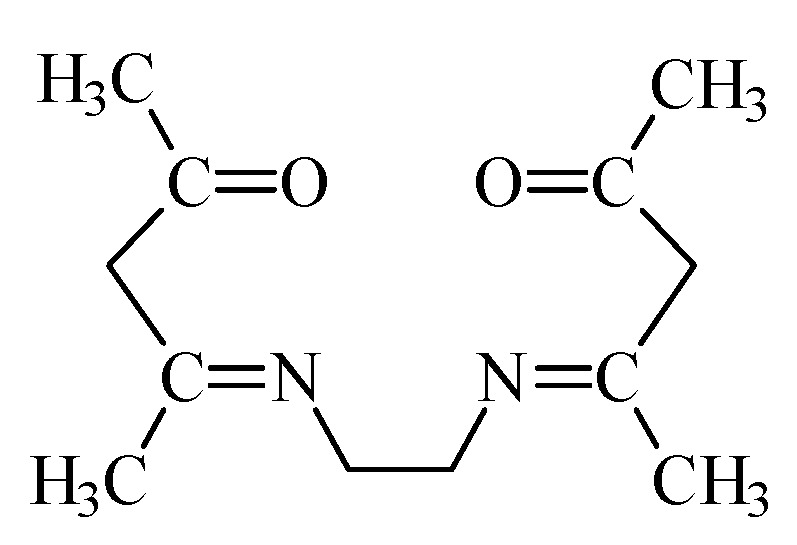
Structure of ethylenodiamino-bis-acetylacetone (EDAB-acac).

Ethylenodiamino-bis-acetylacetone (m.p. 110–111 °C) (Figure 2) was synthesized as a result of the condensation reaction of equimolar amounts of ethylenediamine with acetylacetone according to the procedure described in paper [38]. The structure has been confirmed in the nuclear magnetic resonance studies. The results are given in reference [38].

### 2.2. Extraction and Stripping

SX was carried out in the classical way at 20 °C. Both phases are shaken (volume ratio of aqueous (A) and organic phases (O) A/O = 1) for a period of time (from 1 to 20 min), after which the phases were separated. The organic phase was stripped with water, 3 M HCl, 3 M HNO_3_, 0.5 M NH_3(aq)_, 0.1 M thiourea in 0.1 M HCl and also 0.5 M HCl (A/O = 1).

The atomic absorption spectroscopy (AAS 240FS Spectrometer, Agilent, Santa Clara, CA, USA) or microwave plasma–atomic emission spectroscopy (4210 MP AES, Agilent, Santa Clara, CA, USA) was used to determine the concentrations of metal ions. The determination error did not exceed 5%.

When A/O = 1, the percentage extraction (E, %) of each metal ion was calculated on the basis of changes in the concentration of metals in the aqueous phase before ([M]_i_) and after ([M]_aq_) extraction. Distribution ratio (D) is defined as the ratio ([M]_i_-[M]_aq_)/[M]_aq_. Separation coefficient (S_M1/M2_) is defined as the ratio of the distribution ratios of these metals (D_M1_/D_M2_).

## 3. Results and Discussion

### 3.1. Extraction from One-Component System

#### 3.1.1. Effect of Extraction Time

The effect of time on the extraction of metal ions was investigated by the contacting of an aqueous phase containing 1 mM single-component Pd(II), Au(III) or Pt(IV) ions in 0.1 M HCl with an organic phase containing 1 mM EDAB-acac in methylene chloride.

**Figure 3:**
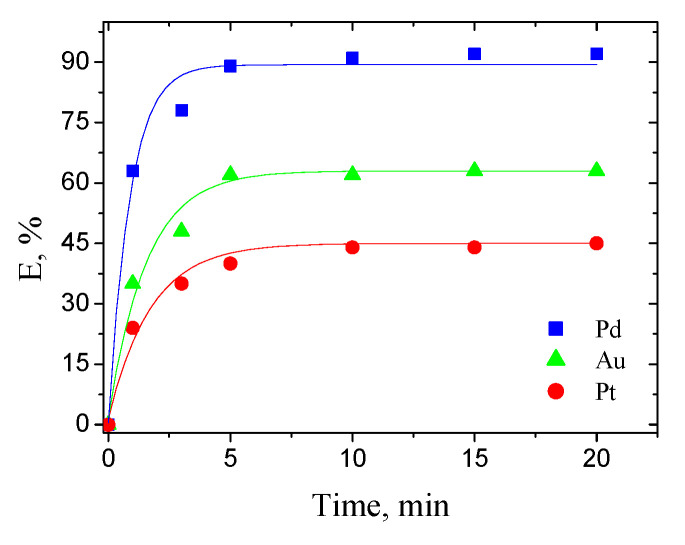
Effect of contact time on extraction of Pd(II), Au(III) and Pt(IV) ions with EDAB-acac in methylene chloride. The initial concentration of each metal ion is 1 mM.

The results are shown in Figure 3.

The extraction process (Figure 3), i.e., the partitioning of the studied metals from EDAB-acac occurs very rapidly. The equilibrium state is reached after only 5 min. In further extraction studies, the phases were separated after 10 min of the process.

#### 3.1.2. Effect of HCl Concentration

**Figure 4:**
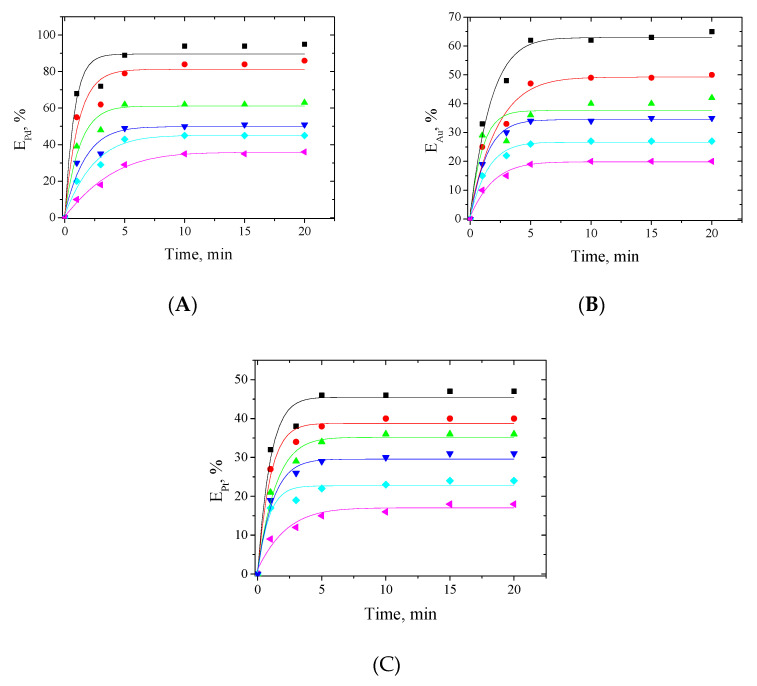
Effect of HCl concentration on extraction of Pd(II) (**A**), Au(III) (**B**), and Pt(IV) (**C**) with EDAB-acac in methylene chloride for ■—0.1; ●—0.5; ▲—1;▼—2; ♦—3; and ◄- 5 M HCl concentration. The initial concentration of each metal ion is 1 mM.

The effect of HCl concentration on the extraction of Pd(II), Au(III) and Pt(IV) with EDAB-acac extractant was investigated and shown in Figure 4. The concentration of HCl in the aqueous phase was altered between 0.1 and 5 M.

The extraction efficiency (%E) of each of the studied metal ions strongly depends on the HCl concentration in the aqueous phase (Figure 4). The highest values of extraction percentage were obtained for 0.1 M HCl. These are 95%, 65%, and 47% for Pd(II), Au(III), and Pt(IV), respectively.

**Figure 5:**
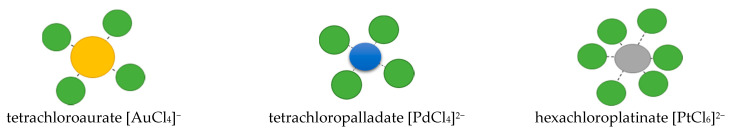
Structure of Pd(II), Au(III), and Pt(IV) chlorocomplexes. Figure was made by authors.

Pd(II), Au(III), and Pt(IV) ions in an aqueous phase at the presence of chloride ions form a series of anionic chloro complexes with the general formula [MCl_n_]^z−^, in which n varies from 1 to N (N—maximum coordination number). In the studied HCl concentration range (0.1–5 M), Pd(II) and Au(III) ions form 4-coordinated, square planar complexes with the formulas [PdCl_4_]^2−^ and [AuCl_4_]^−^, respectively, while Pt(IV) ions form 6-coordinated octahedral complexes ([PtCl_6_]^2−^) (Figure 5) [39,40].

Increasing the HCl concentration in the aqueous phase decreases the extraction percentage of each cation. Perhaps the cause is the competitive ion pair formation reaction [HEDAB-acac]^+^Cl^−^ or [H_2_EDAB-acac]^2+^2Cl^−^.

For 5 M HCl, the extraction percentages are only 36%, 20%, and 18% for Pd(II), Au(III), and Pt(IV), respectively.

#### 3.1.3. Equilibrium of Pd(II), Au(III), and Pt(IV) Extraction

**Figure 6:**
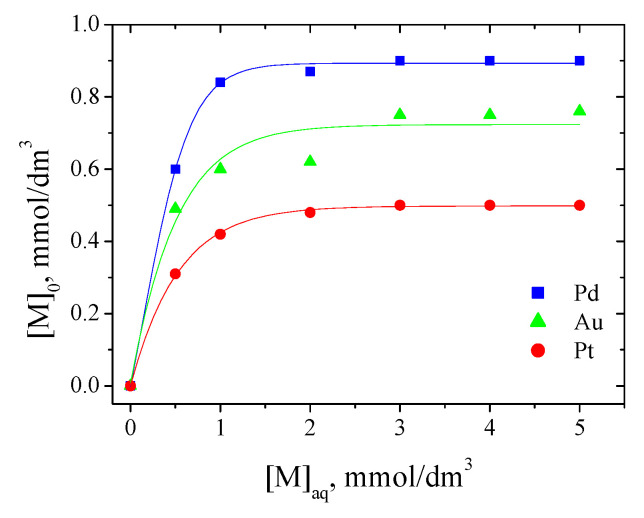
Extraction isotherms of Pd(II), Au(III), and Pt(IV) ions with EDAB-acac in methylene chloride. Initial concentration of each metal ions in the range of 0.5 and 5 mM.

The extraction isotherms of Pd(II), Au(III), and Pt(IV) from a 0.1 M HCl solution using EDAB-acac in methylene chloride are shown in Figure 6. The concentration of metal ions was altered between 0.5 and 5 mM, while the amount of EDAB-acac was fixed (2 mM).

As can be seen from Figure 6, the maximum EDAB-acac capacities for Pd(II), Au(III), and Pt(IV) are 0.9 mM, 0.75 mM, and 0.5 mM, respectively.

The extraction process can probably be described by the following equations:

L_(org)_ + 2H^+^ + 2Cl^−^ = [(LH_2_)^2+^2Cl^−^]_(org)_ (1)

[PdCl_4_]^2−^ + [(LH_2_)^2+^2Cl^−^] = [(LH_2_) PdCl_4_] + 2Cl^−^ [PtCl_6_]^2−^ + [(LH_2_)^2+^2Cl^−^] = [(LH_2_) PtCl_6_] + 2Cl^−^ [AuCl_4_]^−^ + [(LH_2_)^2+^2Cl^−^] = [(LH_2_) (AuCl_4_)_2_] + 2Cl^−^ (2)

According to the above equations, the extractant (L) (EDAB-acac) is first protonated in an acidic solution. Metal ions in HCl medium form an anionic chlorocomplex, which is extracted into the organic phase according to the ion exchange mechanism. This mechanism is consistent with that of Pd(II) extraction with amines [41,42,43].

#### 3.1.4. FT-IR Analysis of the Methylene Chloride Phase

Spectrophotometric studies of the organic phase (methylene chloride) were carried out using a Bruker INVENIO R infrared spectrophotometer (Ettlingen, Germany) equipped with a broadband BeamSplitter and an ATR Quest attachment from Specac (Orpington, UK). The study was carried out in the wave range from 4000 cm^−1^ to 450 cm^−1^.

**Figure 7:**
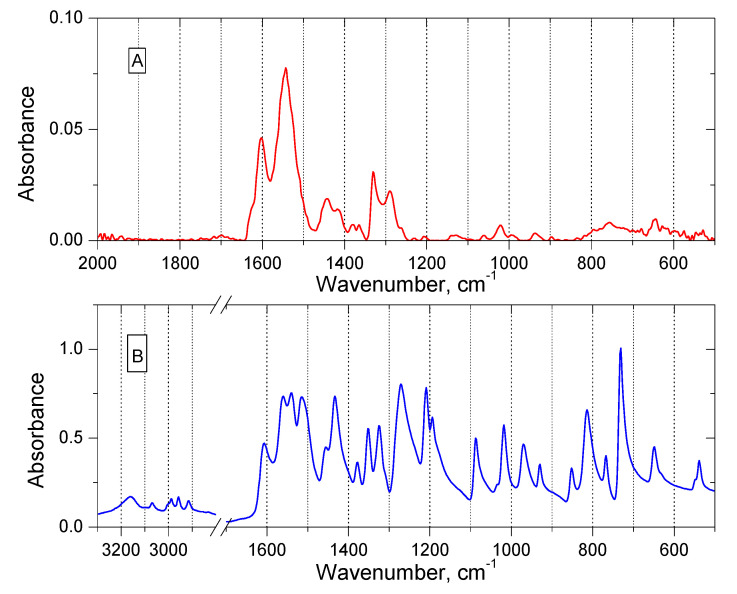
FT-IR spectra of the Pd(II)-chloro-EDAB-acac salt (**A**) and EDAB-acac (**B**) solution in methylene chloride.

FT-IR spectra were performed for an organic phase (methylene chloride) containing EDAB-acac and PdCl^4−^ ions (molar ratio 1:1). The spectrum is shown in Figure 7 together with a spectrum for a solution containing only the ligand (extractant).

IR experiments were interpreted using the IRPal 2.0 software. As can be seen in Figure 7, FT-IR measurements confirm that Pd(II) is transported into the organic phase by the interaction between the extractant (EDAB-acac) and the PdCl_4_^2−^ ions, probably as a [(H_2_EDAB-acac) PdCl_4_]. In the extractant spectrum, the signals corresponding to the C = O and C = N bonds are located at 1606 cm^−1^ and 1560 cm^−1^, respectively. The wide signal within the range of 3150–2990 cm^−1^ corresponds to the stretching vibration in the O-H group formed by the enol form of the extractant. Signals within the range of 1510–1430 cm^−1^ and 1430–1090 cm^−1^ corresponded to deformation N-H and stretching vibrations C-O, respectively. The intense bands were observed in the range of 910–970 cm^−1^ (vibrations of the CH_2_ group) and at 770 cm^−1^ (vibrations C-C). The interaction with Pd(II) caused changes in spectral regions. Namely, changes in the intensity of the vibrational bands C = O (at 1600 cm^−1^), C = N (at 1540 cm^−1^) were observed. The bands stretching both the O-H bonds of the enol form (around 3000 cm^−1^) and the C-O bonds were disappearing.

### 3.2. Extraction from Three-Component System

#### Influence of the Diluent on the Extraction Efficiency

Different diluents of various nature were used to examine the solvent influence on Au(III), Pd(II) and Pt(IV) extraction efficiency. Table 4 shows the results for the extraction of metal ions from 0.1 M HCl with EDAB-acac in toluene, chloroform, methylene chloride, and 2-ethylhexanol.

Table 4 shows that Pd(II) and Au(III) ions are most easily extracted from the three-component Pd-Au-Pt mixture by EDAB-acac, as they can form 4-coordinated planar complexes. The efficiency of the process depends not only on the type of metal ion but also on the diluent used. Depending on the type of diluent, the Pd extraction varies from 54% (toluene) to 95% (methylene chloride). The best diluent conducive to extraction is methylene chloride and 2-ethylhexanol. That might be related to the solvation potential of the extractable complexes. The possibility of solvation is related to the values of diluent donor numbers [44,45]. According to Guttman [46], the donor number (DN) of toluene, methylene chloride, chloroform and 2-ethylhexanol was 0.1, 1, 4, and 48, respectively. As a result of solvation, the hydrophobicity of the extractable complexes increases; hence, diluents with high donor numbers increase the extraction of Pd and Au as well as increase the separation coefficients of Pd in relation to Au and Pt (Table 4).

### 3.3. Stripping Experiments

The stripping of Au(III), Pd(II), and Pt(IV) from the EDAB-acac-methylene chloride phase was investigated. Aqueous solutions like 0.5 M aqueous ammonia, 0.5 M NH_4_SCN, 5 M HCl, 5 M HNO_3_, 0.1 M thiourea in 0.1 M HCl and 1.0 M HCl and also distilled water were used as stripping reagents.

The data obtained for stripping solutions studied are summarized in Table 5.

The stripping with distilled water is not possible. The most efficient stripping of Pd(II) ions was achieved using 0.5 M NH_3aq_, 0.1 M thiourea in 0.1 M and also in 1.0 M HCl, and the percentage of Pd(II) stripping was 100%. The use of 0.5 M NH_4_SCN allow for the recovery of Pd(II) 96% in a single-step process. 5 M HNO_3_ quantitatively strips Pt(IV) ions while 0.1 M thiourea in both 0.1 M and 1.0 M HCl were complete stripping Au(III) ions.

The metal ions can be separated from the Pd-Au-Pt mixture via a 3-step stripping process. In the first stripping step, the organic phase is treated with 5 M HNO_3_ (Pt separation), followed by the use of 0.5 M aqueous ammonia (Pd separation) and, finally, 0.1 M thiourea in HCl (completely stripped Au). In the first proposed stripping step, nevertheless, 45% Pd(II) would be stripped together with Pt(IV),.

### 3.4. Recovery of Pd(II), Au(III), and Pt(IV) from Model Waste Solution

The next step of our work was to investigate the possibility of selective separation and recovery of palladium, gold, and platinum from the model solution with the composition corresponding to the composition of the solution after leaching of the spent e-waste. According to Lu and Xu [9], a typical solution after leaching e-waste with hydrochloric acid contains 10 mg/dm^3^ Pd, 8 mg/dm^3^ Pt, and 10 mg/dm^3^ Au. For the next experiment, a 3-component solution was prepared with the composition given in the literature [9] and 0.1 M HCl was added. Metal ions were extracted with a solution of EDAB-acac in methylene chloride. After 10 min, phases were separated and the concentrations of the Pd(II), Au(III), and Pt(IV) ions remaining in the aqueous phase were determined. The stripping of Au(III), Pd(II), and Pt(IV) from the organic phase was studied with 0.1 M thiourea in 0.1 M HCl. Table 6 shows the extraction efficiency (E,%) and the stripping percentage of Au(III), Pd(II), and Pt(IV) from the e-waste model solution.

The data in Table 6 shows that only Pd(II) and Au(III) ions are extracted. Pt(IV) ions remain in the water phase. The stripping solution allows for the recovery of 90% of Pd(II) and 94% of Au(III). The EDAB-acac can be used as the extractant for the separation of Pd(II) and Au(III) ions from leach solutions of e-waste.

## 4. Conclusions

The conducted research shows that a new amine derivative of β-diketone (EDAB-acac) can be successfully used in an acidic medium to separate a mixture containing Pd(II), Au(III) and Pt(IV) ions by an extraction method. Increasing the HCl concentration decreases the extraction percentage of each ion. Perhaps the cause is the competitive ion pair formation reaction [HEDAB-acac]^+^Cl^−^ or [H_2_EDAB-acac]^2+^2Cl^−^. The highest values of extraction percentage were obtained for 0.1 M HCl. For single solutions of Pd(II), Au(III), and Pt(IV) ions, they are 95%, 65%, and 47%, respectively. The extraction efficiency with EDAB-acac is lower from a 3-component mixture of Pd-Au-Pt in 0.1 M HCl medium. Pd(II) (87–96%) and Au(III) (56–62%) ions that form 4-coordinated planar complexes ([PdCl_4_]^2−^ and [AuCl_4_]^−^) are the easiest to extract. The efficiency of the process depends not only on the metal ion but also on the used diluent. Diluents with high donor numbers increase the hydrophobicity of the extracted complexes due to solvation, thus increasing both the extraction percentage of Pd and Au as well as the separation coefficients of Pd relative to Au and Pt.

Palladium(II), gold(III), and platinum(IV) can be successfully separated from the Pd-Au-Pt mixture in a 3-step stripping process. In the first stripping step, the aqueous phase should be treated with 5 M HNO_3_ (Pt and 45% Pd separation), followed by the application of 0.5 M aqueous ammonia (Pd separation), and, finally, 0.1 M thiourea in HCl (Au separation).

In an acidic medium, the EDAB-acac can be used as an extractant for the separation of Pd(II) and Au(III) ions from e-waste leach solutions.

## Figures and Tables

**Table 1:** Metal content in selected electronic devices, based on available online references [8].

Type	Content, %			Content, % × 10^−3^		
	Fe	Al	Cu	Ag	Au	Pd
CD	7	5	18	280	20	10
Mobile phone	7	3	13	900	200	80
DVD player	62	2	5	115	15	4
Calculator	4	5	3	260	50	5

**Table 2:** The typical composition of basic metals in PCBs, based on available online references [1].

Name	Fe	Al	Cu	Pb	Zn	Sn	Ni
Concentration wt, %	20.47	14.17	6.93	6.30	2.20	1.01	0.85

**Table 3:** Typical composition of valuable elements in PCB, based on available online references [1].

Name	Ag	Ti	Ta	Co	Sb	Cd	As	Au	Se	Ge	Ga	Pd	Pt	Ni
wt, % × 10^−3^	18.9	15.7	15.7	15.7	9.4	9.4	6.3	1.6	1.6	1.6	1.3	0.3	0.2	0.2

**Table 4:** Extraction of mixture Au(III), Pd(II), and Pt(IV) with EDAB-acac in different diluents from acidic medium (0.1 M HCl). The initial concentration of each metal ion was 1 mM.

Metal Ion	Dilutent	E, %	*D_M_*	Separation Coefficient Pd/Au Pd/Pt	
Pd(II)	toluene	54	1.2	2.4	12.0
Au(III)		35	0.5		
Pt(IV)		12	0.1		
Pd(II)	chloroform	87	6.7	5.2	13.4
Au(III)		56	1.3		
Pt(IV)		31	0.5		
Pd(II)	methylene chloride	95	10.1	7.2	12.6
Au(III)		59	1.4		
Pt(IV)		44	0.8		
Pd(II)	2-ethylhexanol	93	13.3	8.3	19.0
Au(III)		62	1.6		
Pt(IV)		40	0.7		

**Table 5:** The metal ion stripping percentages obtained for the stripping solutions studied after EDAB-acac in methylene chloride extraction.

Stripping Solution	Stripping Percent, %		
	Pd	Au	Pt
Water	0	0	0
0.5 M ammonia aq.	100	0	0
0.5 M NH_4_SCN	96	10	63
5 M HCl	70	2	32
5 M HNO_3_	45	0	99
0.1 M thiourea in 0.1 M HCl	100	100	20
0.1 M thiourea in 1.0M HCl	100	100	55

**Table 6:** The extraction efficiency (E,%) and the stripping percent of Au(III), Pd(II), and Pt(IV) from the e-waste model solution. Initial concentration of metals: 10 mg/dm^3^ Pd, 8 mg/dm^3^ Pt, and 10 mg/dm^3^ Au.

Metals	Pd(II)	Pt(IV)	Au(III)
Extraction E, %	82	2	60
Stripping percent, %	90	-	94

## Data Availability

Not applicable.
